# Supplementary material for: TIRAP/Mal Positively Regulates TLR8-Mediated Signaling via IRF5 in Human Cells
Source: Biomedicines. 2022 Jun 22;10(7):1476. doi: 10.3390/biomedicines10071476 (PMC9312982; doi:10.3390/biomedicines10071476)
Supplement: Supplementary file 1 [file biomedicines-10-01476-s001.zip › biomedicines-1747072-supplementary.pdf]

Supplementary Materials

1. Supplementary Materials and Methods

siRNA Treatment THP-1 Cells

For the THP-1 cells, cells were seeded in six-well plates (NUNC, ThermoFisher Scientific, Waltham, MA, USA) at 0.4 x 10^6 per well in antibiotic-free media supplemented with 60 ng/mL of Phorbol 12-myristate 13-acetate (PMA) (Sigma, Merck, Darmstadt, Germany). siRNA transfection (same as listed in the main text) with a final concentration of 16 nM in the well was performed as one-step transfection within 24 h after seeding the cells. Media were changed to PMA-free media 48 h after transfection, and cells were kept for another 48 h before being stimulated by LPS.

TIRAP CRISPR/Cas9 KO Cells Generation

To make the TIRAP KO THP-1 cell line, LentiCRISPRv2 plasmid [24] (gift from Feng Zhang lab - #52961 Addgene, Watertown, MA, USA) was ligated with 5'-CACCGCGTCTGCGTGTGCCACAGTG-3' and 5'- AAACCACTGTGGCACACGCA-GACGC-3' to target the TIRAP gene in exon 5. Packaging plasmids pMD2.G and psPAX2 were used for producing lentivirus (kindly provided by TronoLab, #12260, #12259 plasmids from Addgene, Watertown, MA, USA). HEK293T cells (ATCC, Manassas, VA, USA) were co-transfected with the packaging and lentiCRIS-PRv2 plasmids and washed after 16 h. The lentivirus-containing supernatants were collected after 48 h and used for the transduction of THP-1 WT cells along with protamine sulphate (8 μg/mL final concentration). Transduced THP-1 cells were then selected with puromycin (1 μg/mL) for 2 weeks (early KO) or 1 month (late KO) and tested for TIRAP protein expression with Western blot. All cell lines were regularly checked for mycoplasma contamination.

Phos-tag$_{TM}$ Gel

For total IRF5 phosphorylation analysis, SuperSep Phos-tag gels (50 mol/L, 7.5%, 17 well, 100 × 100 × 6.6 mm) (FUJIFILM Wako Pure Chemical Corporation, Osaka, Japan)were used with Running Buffer (1× Tris-Glycine with 0.1% SDS). Before transfer, gels were washed three times for with gel running buffer containing 20% methanol and 10 mM EDTA for 20 min and once with the same buffer without EDTA to facilitate the transfer procedure (according to manufacturer recommendations).

Kinase Inhibitors

The kinase inhibitors for IRAK-4 (PF-06426779), p38 MAPK (BIRB 796), IKKβ (IKKII–VIII), and TAK1 (5z-7-oxozeaenol) were purchased from Merck (Darmstadt, Germany).

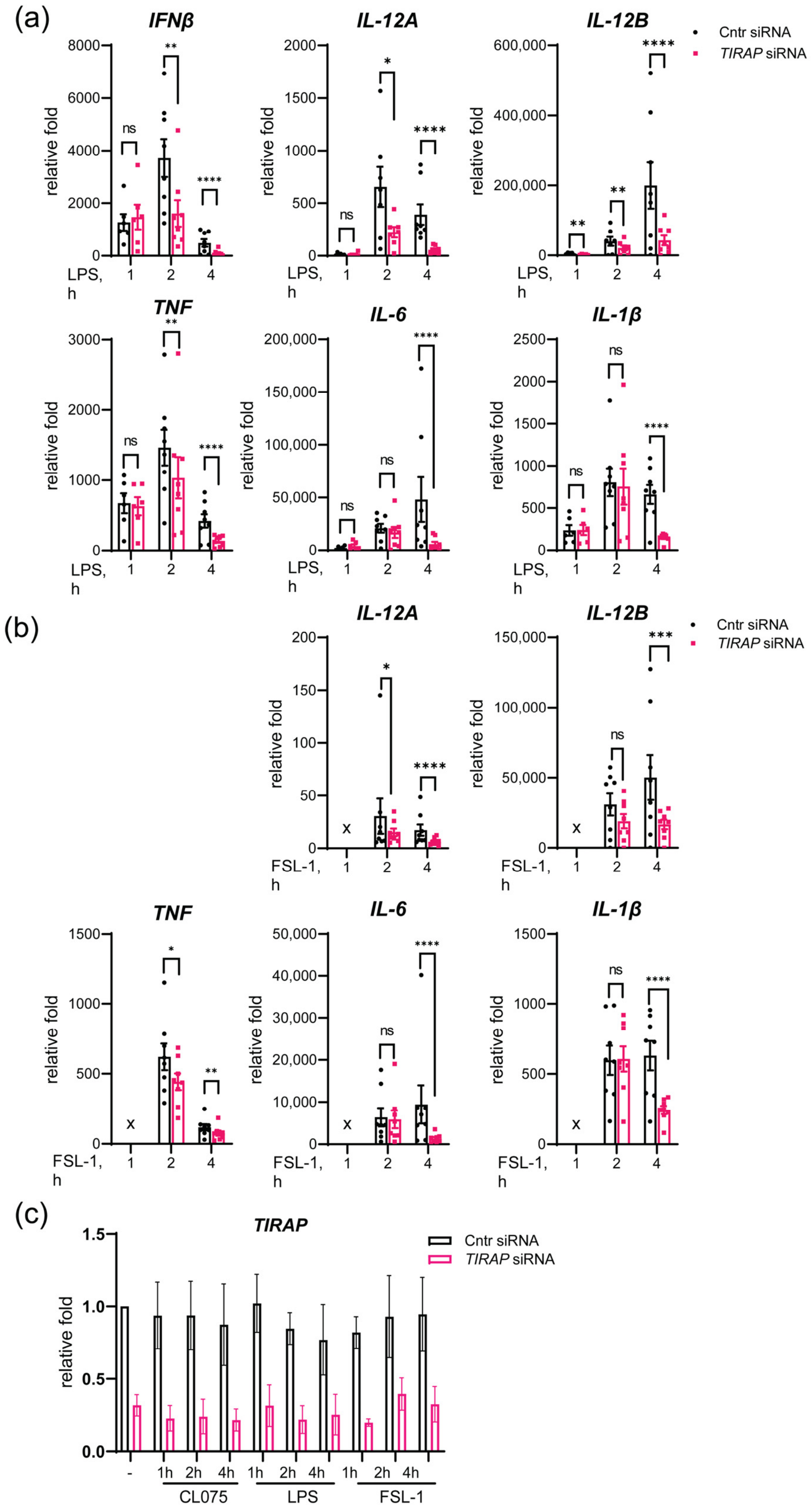

Figure S1. *TIRAP* silencing in primary human MDMs significantly decreases TLR2- and TLR4-mediated cytokine mRNA expression. MDMs were transfected with control or *TIRAP* siRNA followed by stimulation with TLR4 ligand LPS (**a**) or TLR2 ligand FSL-1 (100 ng/mL) (**b**) for the indicated time. RT-

qPCR analysis of cytokine genes expression after stimulation with CL075 in consecutive experiments with cells from different donors (n = 6–8). Data for cytokine expression induced by LPS and FSL-1 stimulation were normalized to the untreated sample and presented as a fold change. Statistical testing was carried out by two-way RM-ANOVA, including a post-test, as described ((*p < 0.05, **p < 0.01, ***p < 0.001, ****p < 0.0001, ns – non-significant)). (**c**) *TIRAP* silencing efficacy in MDMs from all donors was analyzed by RT-qPCR and presented as folds relative to the control siRNA (Cntr) of the non-stimulated MDMs. All graphs and analysis were generated with GraphPad Prism v9.1.2.

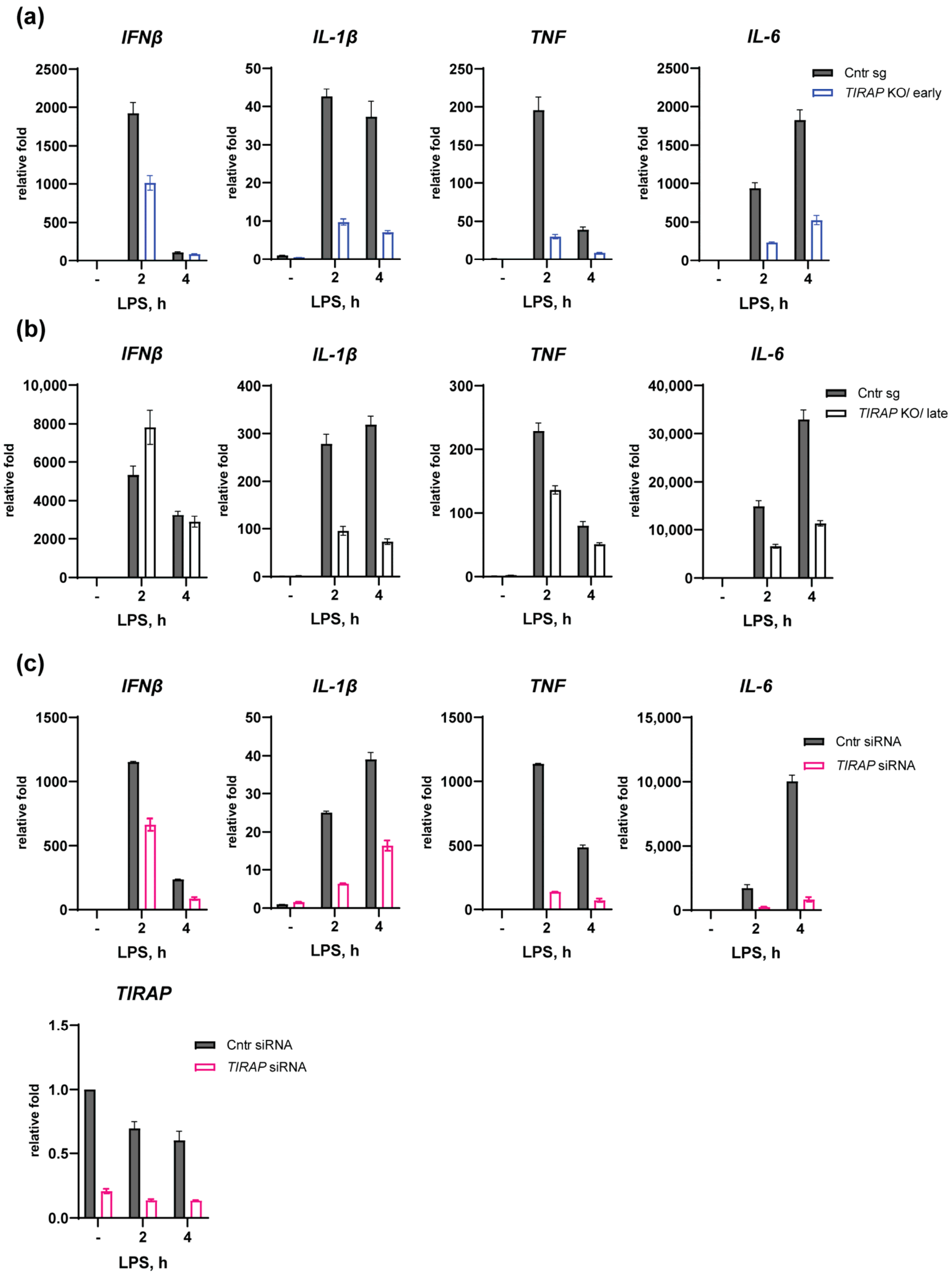

Figure S2. *TIRAP* silencing could be a more relevant approach than knockout to evaluate the fine-tuning of TLRs-mediated signaling by TIRAP. (**a**,**b**) THP-1 cells were transformed by lentiviral vector coding for *TIRAP*-specific or non-targeting control guiding RNA and Crispr/Cas9 to knockout *TIRAP* genes. Cells were selected on puromycin (1 μg/mL) for two (**a**) or four weeks (**b**), corresponding to "early" and "late" *TIRAP* KO. After selection, cells were differentiated by PMA (60 ng/mL) for 24 h, rested for 48 h in PMA-free media, and stimulated by TLR4 ligand LPS (100 ng/mL). (**c**) *TIRAP* was silenced in wild-type THP-1 cells by specific siRNA oligo along with PMA differentiation (72 h with 48 h rest) prior to the addition of LPS. Experiments were performed in two biological replicates followed by RT-qPCR analysis of the cytokines or *TIRAP* expression (**a**–**c**). Data for genes expression were normalized to the untreated samples and presented as fold changes with the mean and SD.

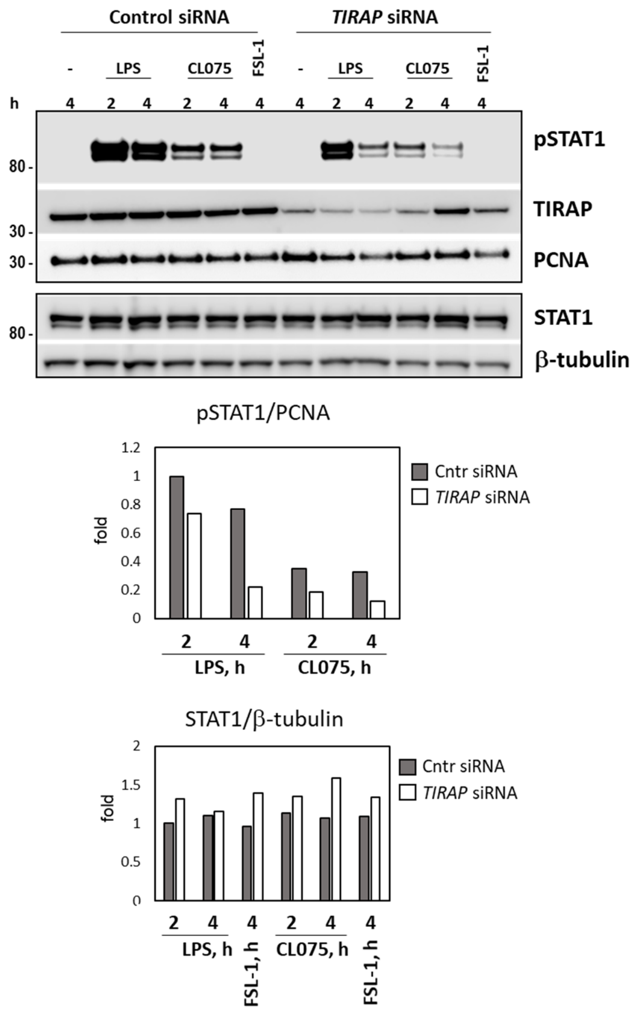

Figure S3. *TIRAP* silencing reduces STAT1 phosphorylation in LPS- or CL075-stimulated human MDMs. Phosphorylation of Y701 in STAT1 protein, total STAT1 protein levels, and TIRAP protein expression after silencing were addressed by Western blot analysis with specific antibodies. Representative images for one of 3–6 donors. Samples represent protein fractions isolated simultaneously with total RNA from Qiazol lysates. Samples from FSL-1-stimulated cells were used as a negative control for the IFNβ-dependent phosphorylation of STAT1. PCNA and β-tubulin Western blotting were performed for the loading control and quantification of pSTAT1, and total STAT1 levels are presented on the graph as a relative fold. Western blots are grouped in frames together with their loading control blot.

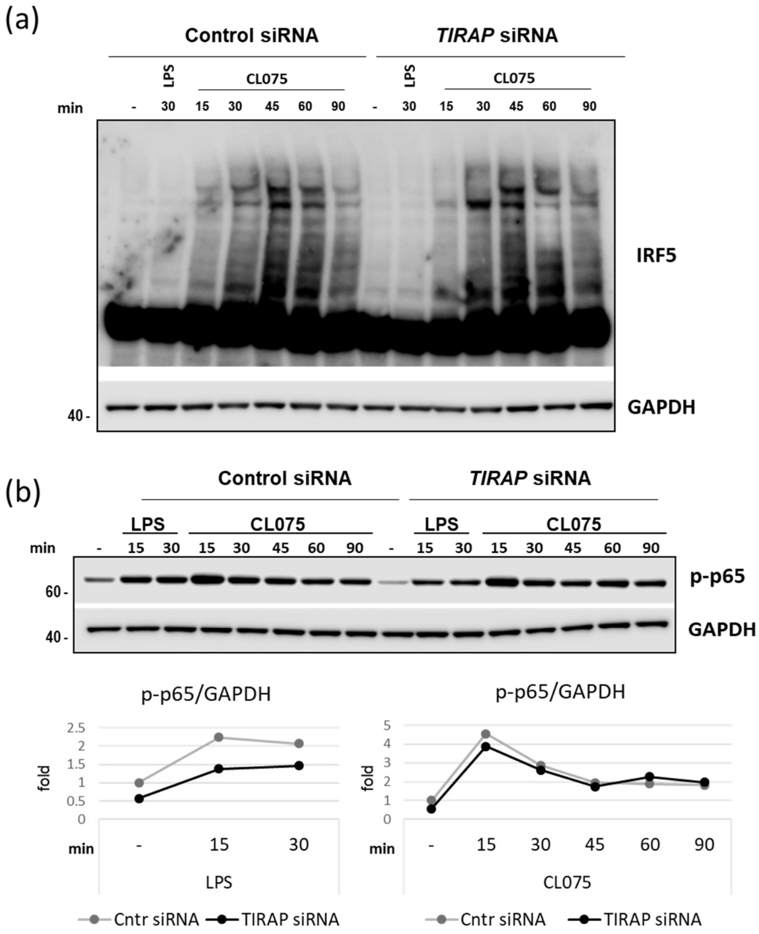

Figure S4. Total phosphorylation pattern of IRF5 and p65 phosphorylation in CL075-stimulated cells were not affected by *TIRAP* silencing much. MDMs were pre-treated by control or TIRAP silencing oligo followed by stimulation with TLR8 ligand CL075 (2 µg/mL) or LPS (100 ng/mL). (**a**) Lysates were resolved using PhosTag gel as recommended by manufacturer followed by Western blot analysis using total IRF5 Abs. GAPDH Western blot (lower panel) after parallel conventional SDS-PAGE was used for loading control. One of two independent experiments. (**b**) Conventional Western blot analysis to address phospho-p65 S536 levels in LPS or CL075 stimulated macrophages silenced by control or *TIRAP* oligo. Representative image and graphs for one of four donors. Densitometry analysis and normalization to loading control GAPDH was carried out using LiCor Odyssey Image studio v5.2 software.

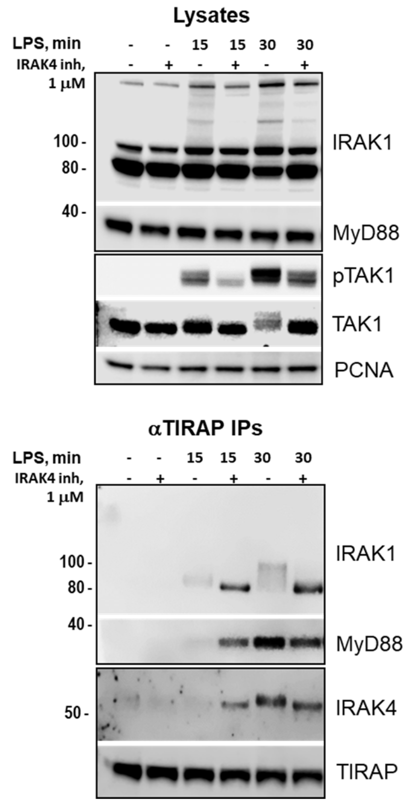

Figure S5. Band shift for IRAK4 protein in TIRAP IPs induced by pre-treatment of cells by PF-06426779 IRAK4 inhibitor supports the specificity of IRAK4 staining for the selected IPs and WB conditions. PMA-differentiated THP-1 cells were pre-treated with DMSO or PF-06426779 IRAK4 inhibitor (1 μM) for 30 min before stimulation with LPS (100 ng/mL). Endogenous TIRAP was immunoprecipitated for four hours as described. Cellular lysates were analyzed in parallel to control for input and efficacy of signaling inhibition with IRAK4 inhibitor. One of two experiments.

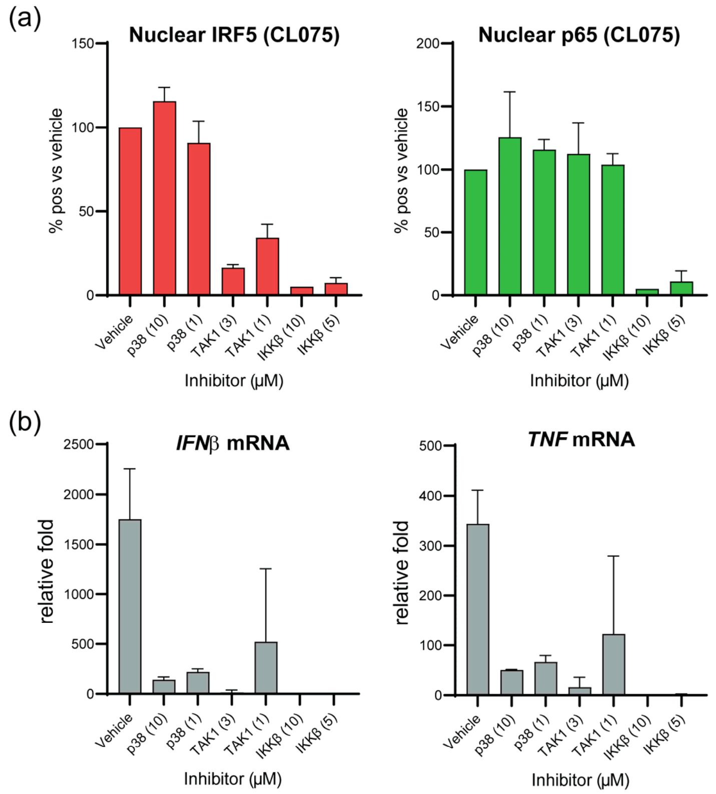

Figure S6. Pharmacological inhibition of p38 MAPK does not block TLR8-induced nuclear translocation of IRF5 or p65 but still suppresses TLR8-induced cytokine transcription. Human monocytes were pre-treated with chemical inhibitors of p38 MAPK (BIRB-796), TAK1 (5z-7-oxozeanol), and IKKβ (IKKII-VIII) for 30 min and subsequently stimulated with CL075 (1 µg/mL). Concentration of inhibitors (µM) shown in brackets on x-axis. (**a**) Activation of IRF5 and p65/RelA was examined after 1 h of stimulation by immunostaining and quantitative imaging of nuclear staining with Scan^R. After treatment with vehicle (0.1% DMSO) and CL075 stimulation, ~30% and ~45% of the cell nuclei stained positive for IRF5 and p65, respectively, while in non-stimulated cells, the corresponding numbers were <5% and <15% (not shown). The frequencies of cells with nuclear staining after treatment with inhibitors and CL075 are normalized against the vehicle control (adjusted to 100%). (**b**) Induction of cytokines in parallel wells treated with inhibitors and stimulated with CL075 for 2 h. *IFNβ* and *TNF* expression was quantified by RT-qPCR. Bars represent mean + SD for consecutive experiments with monocytes from different donors (n = 2).

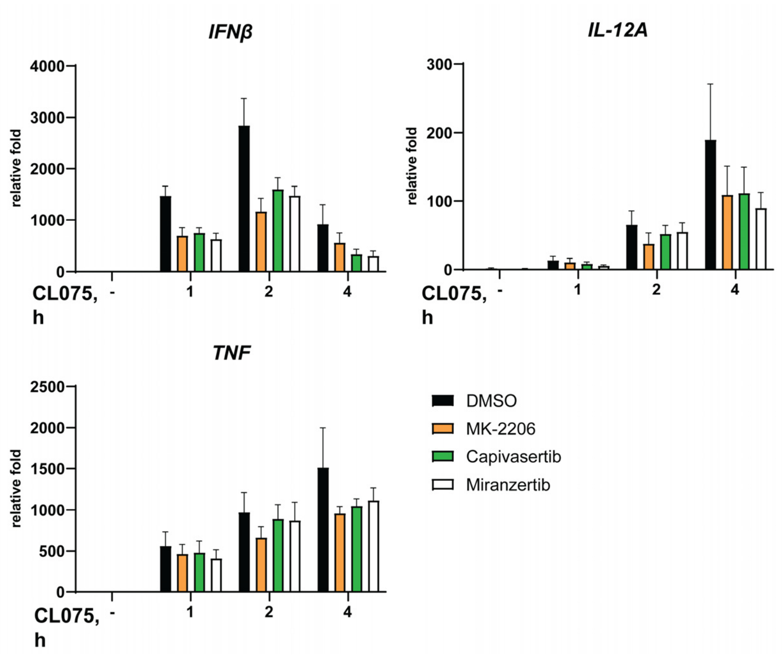

Figure S7. Tested Akt inhibitors have similar inhibitory effect on TLR8-mediated *IFNβ* and *IL-12A* expression. RT-qPCR analysis of cytokine gene expression in MDMs pre-treated for one hour with three specific Akt inhibitors (2 μM) followed by stimulation with CL075 (2 μg/mL) for indicated time. Data for genes expression were normalized to the untreated sample and presented as a fold change. Data presented as mean and SEM for consecutive experiments with MDMs from different donors (n = 3).

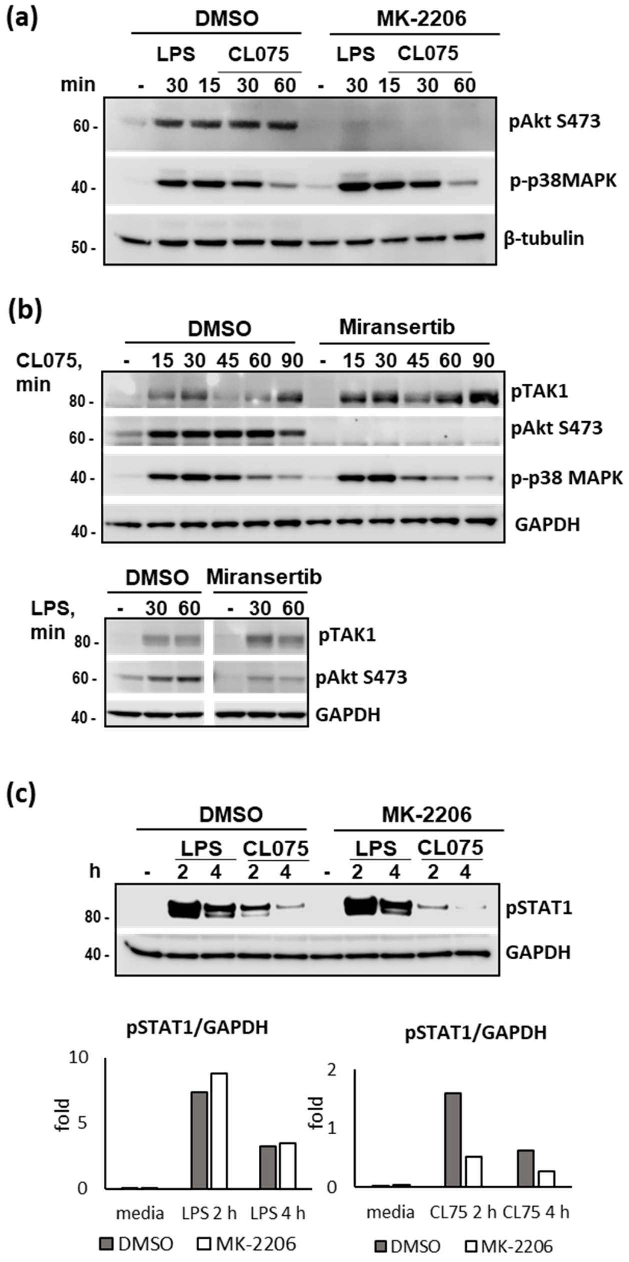

Figure S8. Inhibition of Akt strongly decreases TLR8-mediated phosphorylation of Y701 in STAT1 with no inhibitory effect on TLR4- or TLR8-mediated MAPKs phosphorylation. Western blot analysis of lysates from MDMs that were pre-treated with allosteric Akt inhibitors (**a,c**) MK-2206 or (**b**) Miransertib (2 μM) for one hour and stimulated with 2 μg/mL CL075 or LPS (100 ng/mL). To control for inhibition efficacy, Akt (S473) phosphorylation level was evaluated by Western blot. Cropped images depicting LPS stimulation are from the same membrane with excised and irrelevant bands. GAPDH or β-tubulin were used for equal loading control on the same membrane as grouped blots (**a-c**). Graph (**c**) shows quantification of p-STAT1 levels relative to GAPDH. Quantification of band intensities was carried out using LiCor Odyssey Image studio v5.2 software. Western blots representative of one of four donors.

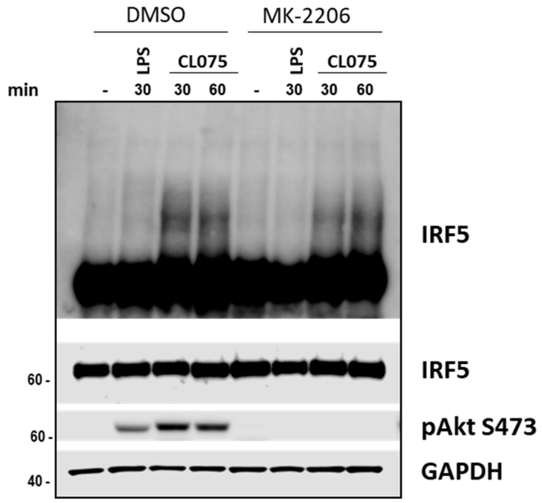

Figure S9. Akt inhibition has no clear effect on the total phosphorylation pattern of IRF5. MDMs were pre-treated with MK-2206 (2 μM) followed by stimulation with CL075 (2 μg/mL) or LPS (100 ng/mL) for indicated time with LPS used as a negative control for IRF5 phosphorylation. Lysates were resolved using PhosTag gel followed by Western blot analysis of total IRF5 (top panel). IRF5 Western blot after parallel conventional SDS-PAGE was used as a loading control (second panel). To control for Akt inhibition efficacy, the Akt (S473) phosphorylation level was addressed with GAPDH for loading control. One of two experiments.
